# Phenotyping Chronic Pelvic Pain Based on Latent Class Modeling of Physical Examination

**DOI:** 10.1155/2013/891301

**Published:** 2013-12-22

**Authors:** B. W. Fenton, S. F. Grey, M. Reichenbach, M. McCarroll, V. Von Gruenigen

**Affiliations:** ^1^Department of Obstetrics and Gynecology, Summa Health System, 525 E Market Street, Akron, OH 44309-2090, USA; ^2^Department of Epidemiology and Biostatistics, College of Public Health, Kent State University, Kent Hall 136C, Kent, OH 44242, USA

## Abstract

*Introduction*. Defining clinical phenotypes based on physical examination is required for clarifying heterogeneous disorders such as chronic pelvic pain (CPP). The objective of this study was to determine the number of classes within 4 examinable regions and then establish threshold and optimal exam criteria for the classes discovered. *Methods*. A total of 476 patients meeting the criteria for CPP were examined using pain pressure threshold (PPT) algometry and standardized numeric scale (NRS) pain ratings at 30 distinct sites over 4 pelvic regions. Exploratory factor analysis, latent profile analysis, and ROC curves were then used to identify classes, optimal examination points, and threshold scores. *Results*. Latent profile analysis produced two classes for each region: high and low pain groups. The optimal examination sites (and high pain minimum thresholds) were for the abdominal wall region: the pair at the midabdomen (PPT threshold depression of > 2); vulvar vestibule region: 10:00 position (NRS > 2); pelvic floor region: puborectalis (combined NRS > 6); vaginal apex region: uterosacral ligaments (combined NRS > 8). *Conclusion*. Physical examination scores of patients with CPP are best categorized into two classes: high pain and low pain. Standardization of the physical examination in CPP provides both researchers and general gynecologists with a validated technique.

## 1. Introduction

Establishing phenotypes for clinical conditions is a fundamental step in the development of diagnostic criteria, which are required for coherent research and evidence based clinical care [1]. From the categorization of fetal heart rate patterns to the description of pelvic organ prolapse, a validated nomenclature allows an apples-to-apples comparison of research studies and also lets clinicians translate research findings into practice by clearly describing a clinical condition in terms of objective findings.

Chronic pelvic pain is an area of gynecology sorely in need of evidence based phenotypes [2]. The current phenotyping approaches are primarily symptom based and limited to urologic pain [3]. The challenges in this field are many and varied [4]. Since pain is subjective, an easily replicated standardized examination becomes even more important. How to perform the exam, which points to examine, and where to set thresholds between incidental pain and significant pain are all problems faced by clinicians on a daily basis [5]. Patients also are frustrated by a lack of uniformity in describing their condition and are hindered by incomplete evaluations [6]. Phenotyping patients into similar groups can be used clinically to assess prognosis, evaluate potential treatments, or suggest further diagnostic evaluation.

To address these concerns requires a substantial sample of patients, an extensive standardized physical examination, fundamental inclusion criteria, and some technique for data reduction. Once such method is known as latent class analysis. In this approach, a large data set is mathematically examined to find related classes of patients hidden (latent) within the data structure. The analysis is stepwise, beginning with an assurance that the columns (in this case physical examination locations) are all appropriately measuring a single construct (i.e., they are unidimensional). Once this is done, the values of the examination sites are evaluated using multiple measures to determine how many latent classes exist in the data set. The number of latent classes present (i.e., 2 classes: high and low; 3 classes such as high, low, and intermediate; or 4 classes: minimal, mild, moderate, and severe) is determined based on several mathematical criteria. Once the number of classes is known, thresholds for class allocation can be created, and then the original larger number of evaluation sites can be reduced to only the most pertinent locations. These can then be used to establish research or clinical phenotypes.

The objective of this study was to apply latent class modeling to the physical examination of patients with chronic pelvic pain, with the ultimate goal of defining clinical phenotypes in these patients.

## 2. Methods

A total of 476 female patients were evaluated following referral to the Pelvic Pain Specialty Center at Summa Health System according to Institutional Review Board-approved protocol 07048. All patients met the American College of Obstetricians and Gynecologists definition for CPP and were evaluated in a standardized manner similar to that suggested by the International Pelvic Pain Society (http://www.pelvicpain.org/resources/handpform.aspx). Patients underwent a structured history including a clinical interview by a board certified psychiatrist and a complete physical examination by a board certified gynecologist, including semiquantitative pelvic pain testing. This was done across multiple sites of the pelvis, including the pelvic abdominal wall, the vulvar vestibule, the pelvic floor, and the vaginal vault.

Patients were placed in the dorsal lithotomy position and pain on the abdominal wall was evaluated with pain pressure threshold (PPT) algometry according to previously described protocols [7]. The physical examination included the application, perpendicular to the abdominal wall, at a rate of approximately 1 kgf/s of a pressure algometer (Wagner Instruments, Greenwich, CT, model FPK 10) with a 1 cm^2^ tip at 14 sites of the lower anterior abdominal wall. Pressure was steadily applied until patients reported a change from pressure to pain. Evaluations were done based on PPT suppression, calculated as the patient's threshold value subtracted from the maximum value of 3 kgf applied.

Pelvic floor pain testing was done using a lubricated, gloved single finger administered by a trained examiner applying 1 kg/cm^2^ of force to the central point of each area [8]. Before testing the examiner reviewed the pressure needed to apply 1 kg/cm^2^ by training on the pressure algometer. No pain was rated at zero, otherwise from one to ten, with ten being coached as “the worst pain imaginable.” The pelvic floor muscles were palpated in order, starting with the puborectalis, palpated in the middle of its body at the 4 and 10 o'clock position from the introitus. The pubococcygeous-iliococcycgeous complex was palpated approximately 2 cm dorsal to this position, in the midbody of the muscle. The obdurator internus was provoked by instructing the patient to adduct her flexed leg against resistance while the muscle body was palpated.

The vulvar skin was tested using a cotton tipped applicator with just enough force to indent the mucosa. This was done in 6 locations in order starting at the 12:00 position and progressing clockwise in the vestibule at the 2, 4, 6, 8, and 10:00 positions. For the vaginal vault, gentle posterior-lateral traction was applied over the uterosacral ligaments bilaterally, and then anterior-lateral traction was applied to the adnexa. Pain from these sites was also recorded on the 0–10 numeric rating scale.

Statistical analysis was conducted in three steps. First, an exploratory factor analysis (EFA) was conducted using the 30 examination sites noted above to test the unidimensionality of each pelvic region, where it was expected that sites from each region would load onto one of four factors representing each region. Geomin rotation, an oblique rotation method that permits factors to correlate, was used as pain within one region is expected to be related to pain in another region [9]. The number of factors extracted was determined from a scree plot of factor eigenvalues to identify the “breakpoint” where the curve flattens out [10, 11]. Evidence of unidimensionality within each region was established by statistically significant item factor loadings with standardized values greater than 0.35 and without substantial cross loadings on other regions. Next, a latent profile analysis (LPA), a type of latent class analysis in which the class indicators are continuous variables like the examination site pain measures used in this study, was then used to classify patients into groups with similar patterns of pain within each pelvic region, using the sites that had loaded onto that region in the EFA [12]. The number of classes for each region was assessed using multiple statistical fit criteria, but primarily determined by the Bayesian Information Criterion (BIC) [11]. Entropy was used as an indicator of how well subjects can be differentiated between classes [13]. Both EFA and LCA were conducted with Mplus version 7 [14]. Last, receiver operator characteristic (ROC) curves were calculated to establish the area under the curve (AUC) for each examination site, which was used to identify the best threshold value for each site and compare the predictive performance of the sites within each region. Bootstrapping with 10,000 samples was used to calculate the confidence intervals of sensitivity and specificity at each threshold value for each site, and to statistically compare ROC curves for each site using the “pROC” package from the R statistical program.

## 3. Results

Patients tolerated the evaluation well, with no more than 5% missing data points. Within a given region the combination of missing data from multiple exam sites slightly reduced number of subjects for each LCA. Demographic characteristics of this population with CPP are shown in Table 1. Exploratory factor analysis of the tested sites is shown in Table 2(a). The EFA with four factors showed the appropriate factor loadings of the examination sites on the four hypothesized areas of abdominal wall, vulva, pelvic floor, and vaginal vault as demonstrated in Table 2(b).

Table 3 demonstrates the results for multiple methods (Log-likelihood, BIC, entropy, and smallest class) for determining the number of latent classes present. Based on the results of Table 2, these are divided into the tests of the abdominal wall sites (Table 3(a)), the vulvar sites (Table 3(b)), the pelvic floor sites (Table 3(c)), and the internal vaginal vault sites (Table 3(d)). For each location, a two-class solution provides an optimized classification scheme based on all the different parameters.

**Figure 1:**
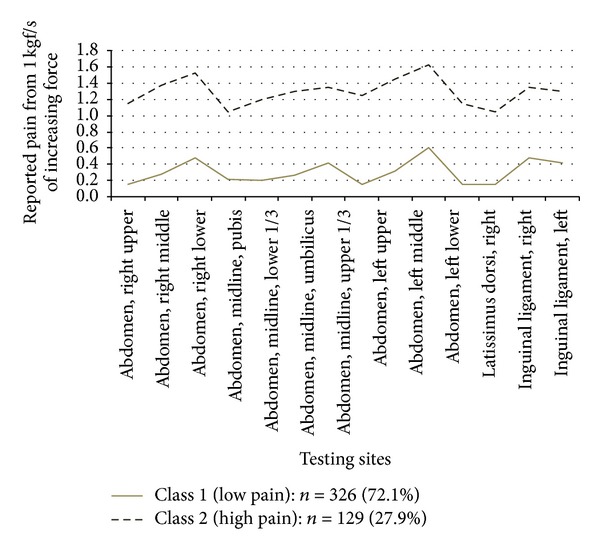
Pain pressure thresholds of 14 abdominal wall testing sites across two latent classes.

**Figure 2:**
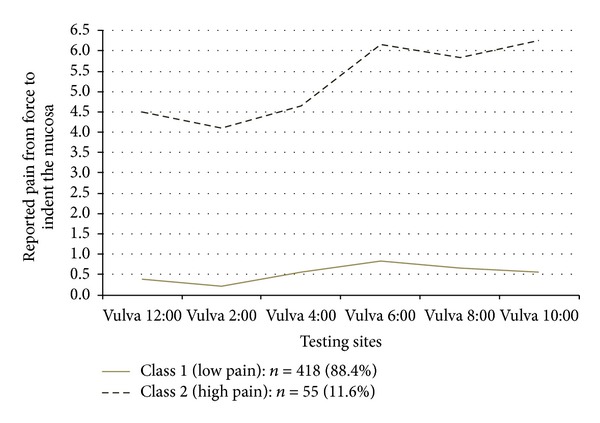
Pain pressure thresholds of 6 vulvar testing sites across two latent classes.

**Figure 3:**
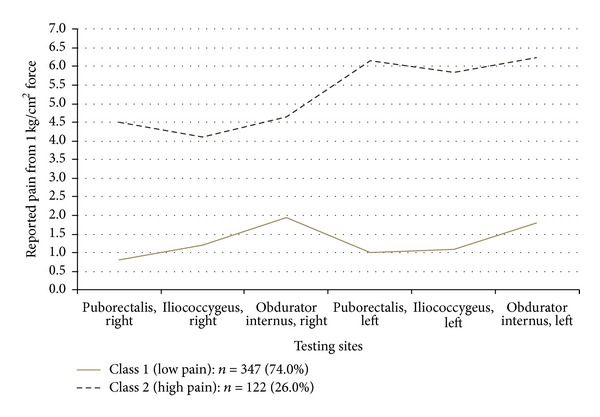
Pain pressure thresholds of 6 pelvic floor muscle testing sites across two latent classes.

**Figure 4:**
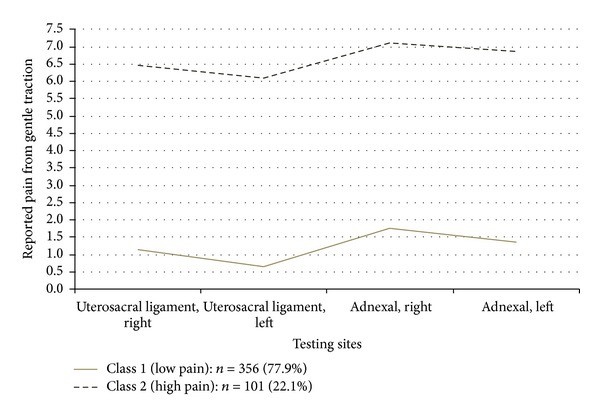
Pain pressure thresholds of 4 vaginal vault testing sites across two latent classes.

The two latent classes for each region determined in Table 3 are depicted graphically in Figures 1–4. Classification of pain pressure threshold suppression of the abdominal wall sites is depicted in Figure 1. Class 1 represents patients with low levels of threshold suppression (i.e., these patients have very low pain in the abdominal wall) and Class 2 includes only patients with a high degree of pain pressure threshold reduction (i.e., they are very tender to touch). On this scale a score of 0 means that the patient can tolerate 3 kg/cm^2^ force applied to the abdomen without reporting any pain, while the maximum score (2.5) means that the patient reports a sensation of pain (rather than pressure) with only 0.5 kg/cm^2^ force applied at that point (the equivalent of light touch). Thus patients with low thresholds have greater pain sensitivity (Class 2) and patients with high PPTs do not report a feeling of pain until a substantial pressure is applied (Class 1).

Classification of the numeric ratings for pain with light touch to the vulvar vestibule is shown in Figure 2. Class 1 represents patients with little or no pain, and Class 2, represents patients reporting high levels of pain to light touch.  Figure 3 demonstrates the classification of patients based on pain scores in the pelvic floor muscles. Class 1 patients report low levels of pain on palpation, and Class 2, report high levels of pain. Classification of patients according to palpation of the vaginal vault is shown in Figure 4. Class 1 patients report low levels of pain on palpation, and Class 2 report high levels of pain.

In Table 4 the examination sites (paired) are compared to the 2 class solution using receiver operator characteristic curve analysis to determine the site most predictive of class membership. Table 4(a) demonstrates results for the abdominal wall sites, with the left and right middle abdomen having the greatest area under the curve (AUC) with a pain pressure threshold suppression of 2 or more to be included in the high pain class (class 2). In this analysis, any sum of values for these two sites (i.e., 0.5 on the right and 1.5 on the left) would result in that patient being included in the high pain class. Testing sites on the lateral abdominal wall (between the iliac crest and lower costal margin) and the inguinal ligaments are significantly worse than the best pair (left and right middle abdomen) for assignment of patients into a high or low pain class.

Table 4(b) demonstrates the results of ROC testing based on the two class solution for the vulvar pain sites. A report of pain of 2 or more at the 10:00 position in the vestibule is enough to include the patient in the high pain class. Examination at the 12:00, 2:00, and 4:00 positions are significantly less accurate at assigning patients into the two class solutions.

Table 4(c) demonstrates the results of an evaluation of the pelvic floor muscle sites. A summed report of pain of 6 or more at the left and right puborectalis classifies a patient into the high pain class. These examination sites are all statistically equivalent to each other, but to be classified as high pain, the sum of the reports of pain at the obdurator internus is higher, 10 or more (out of a maximum of 20 for any sum).

Classification of patients based on testing the uterosacral ligaments or adnexal tenderness is demonstrated in Table 4(d). A summed report of pain of 8 or more on the uterosacral ligaments classifies a patient into the high pain class. Pain in the adnexa is significantly less accurate in classifying patients.

## 4. Conclusion

This approach to phenotyping represents a combination of quantitative and semiquantitative methods suitable for both research and clinical application. The mathematical approach taken here is a stepwise method, assuring that the examination sites in the different regions tested actually measure one construct (unidimensionality: Table 2), determining how many latent classes are present in the data (based on multiple statistical fit criterion: Table 3), applying the classification scheme to the physical exam data (Figures 1–4), and then determining the optimal examination sites and thresholds for class assignment (ROC analysis: Table 4).

Quantification of the pelvic pain examination was performed according to previously published protocols using little or no instrumentation. This approach has the significant advantage that it can then be widely applied to clinical practice or deployed to multiple research sites with a limited equipment cost. A wide range of alternative techniques are available, using thimble algometers or other custom crafted devices [15]. Further research may demonstrate these to be excellent research tools, but until they are widely available these techniques have limited applicability in creating clinical phenotypes for use by typical clinicians.

One benefit of this approach is to highlight the complexity of the pelvic pain evaluation. Chronic pelvic pain is a significant problem for a substantial proportion of our patients and demands more than just a cursory bimanual examination. Although many pain diagnoses may be present which are not easily determined on physical examination, at a minimum this study indicates that pain in the abdominal wall, vulvar vestibule, pelvic floor, and uterosacral ligaments should be evaluated separately.

Setting thresholds and standardizing the examination are vital milestones for phenotyping clinical conditions. Chronic pelvic pain is an excellent example of complex disease, with multiple purported risk factors [16], heterogeneity within the tissues [17], heterogeneity within an individual diagnosis [18], and the complexity of multiple diagnoses potentially present in a single individual patient [19]. Determining useful sites to examine, the number of classifications existing at each examination point, and the thresholds for assigning classification are all vital steps toward developing evidence based phenotypes for use in research and clinical practice.

The results reported here offer some unique insights into the structure of pain related diagnoses. Although many scales rate pain along a continuum (such as the PROMIS approach [20], or the VAS pain scale), all four of the regions evaluated here produced two latent classes: high pain and low pain. There was no a priori determination of how many classes might exist hidden in the data. Since all patients met ACOG criteria for CPP and were being seen in a referral center, it was felt possible that only one class would be found (everyone would have significant pain). Similarly any number of classes could be postulated: low, medium, and high pain (3 classes), or 5 classes in a Likert style scale. Based on these results, future studies of a general clinical population should report physical examination pain as none, low, or high pain. Studies of pain populations should report their exam findings as high or low pain.

This study has a number of methodological limitations which must be acknowledged and which will need to be addressed by further study. The examination is conducted by a single clinician with experience in evaluation of chronic pelvic pain. Although this technique is largely replicated in other studies using multiple examiners at multiple institutions [21], these studies are also performed by clinicians with a special interest in CPP. The generalizability of these results to screening populations of patients without CPP or to other examiners will similarly require further study. As a study of female chronic pelvic pain, it is possible but unknown to what extent these conclusions can be transferred to men with chronic pain.

Other limitations include the analysis of a finite number of examination sites. A wide range of other sites to test exist in the pelvis, which include the adductors, pyriformis, and coccygeus among others. Inclusion of other examination sites has the potential to provide better correlation with the different classes; however Table 4 demonstrates that there are limited (though sometimes statistically significant) differences between the sites tested here. Based on this finding, the addition of other sites in similar regions of the pelvis is unlikely to alter the fundamental findings of this study but may produce alternative examination sites with different thresholds.

The evaluation of pelvic pain in this study followed a prescribed protocol which was followed for all patients. This approach has the advantage of producing a complete data set, but the disadvantage of introducing bias based on the order of testing. This is most prominently displayed in evaluation of vulvar pain. In this study the order of examination began at the 12:00 position and then proceeded in a clockwise manner to end at the 10:00 position. Since pain can be worsened through sensitization due to previous stimulation (examination) of nearby sites, it is possible that the increased AUC at the 10:00 position is an artifact of the order of examination. Further research with a randomized vulvar testing scheme may reveal a different threshold or point for assignment to the high pain class.

This study represents an effort to produce a statistically sound approach to phenotyping CPP arising from the abdominal wall, vulva, pelvic floor, and vault; it is not designed to evaluate any of the other myriad disorders associated with CPP. In particular pain arising from the uterus, which may be due to a number of conditions including fibroids, endometriosis, or adenomyosis, is excluded. Evaluation of pain of this type demands histologic correlation and is beyond the scope of this study. Similarly pain from the bladder (which may be associated with interstitial cystitis or merely a bladder infection) and pain from the rectum (which may be due to irritable bowel syndrome, diverticular disease, or hemorrhoids) are also excluded intentionally from this evaluation. The rationale for this is explored in a separate latent class analysis of these pelvic floor locations [8].

This study is clearly a beginning rather than an end in itself. With a phenotyping methodology is available, many future possibilities exist. In particular, a physical exam based phenotype can be applied to clinically defined disorders such as endometriosis and interstitial cystitis to determine the different subpopulations within these diagnoses. A more precise classification scheme in chronic pain states has the potential, with future research, to assist clinicians with an optimized selection of treatments and can provide patients with a more clear prognosis based on the results of standardized outcomes.

### 4.1. Clinical Implications

Based on these results, four CPP phenotypes can be defined.

Pain in the anterior abdominal-pelvic wall can be separated into two classes, with high pain defined when PPT depression in the left and right midwall (measured halfway between the level of the umbilicus and the inguinal canal at the lateral border of the rectus muscle) equals a sum of 2 or more. Other abdominal wall locations can also be used, including the midline, upper, or lower lateral rectus borders, but with different thresholds. The lateral abdominal wall or inguinal ligaments are not as accurate in classifying patients based on abdominal-pelvic wall pain.

Pain on the vulva can be can be separated into two classes, with high pain defined based on a report of pain with gentle mucosal indentation of the vestibule at the 10:00 position producing a reported pain of 2 or more. The 6:00 and 8:00 positions can also be used, with different thresholds. The 12:00, 2:00, and 4:00 positions are not as accurate in classifying patients into high and low vulvar pain groups.

Pain in the pelvic floor can be separated into two classes, with high pain defined based on a report of pain on palpation of the left and right puborectalis with a sum of reported pain of 6 or more. The obdurator internus and iliococcygeus can also be used to classify patients but with different thresholds.

Pain in the vaginal vault can be separated into two classes, with high pain defined based on a report of pain on palpation of the left and right uterosacral ligaments with a sum of reported pain of 8 or more. Pain in the adnexa is not as accurate in classifying patients into high and low pain groups.

## Figures and Tables

**Table 1:** Demographic Characteristics of 476 Women Presenting at the Pelvic Pain Specialty Center.

Characteristic	*n*/mean	%/SD
Age in years	34.9	10.4
Education:		
College degree	189	40%
High school	212	45%
Less than high school	49	10%
Unknown	26	5%
Insurance status:		
Unknown	15	3%
Private	189	40%
Public	172	36%
Charity	100	21%
White race	370	82%
Months of pain	51.3	56.6
Gravidity	2.3	2.2
Parity	1.5	1.4
Childhood trauma score	4.1	5.2
Catastrophization score	27.5	14.2
PROMIS subscale scores:		
Anger	53.9	10.5
Anxiety	55.3	10.5
Depression	53.3	10.9
Fatigue	56.9	9.2
Pain Behavior	61.1	6.8
Pain Impact	62.9	8.3
Physical function	42.3	8.6
Social activity	45.0	8.7
Social role	41.6	9.4
Sleep disturbance	58.4	9.8
Wake disturbance	55.9	10.1

**Table:** (a)

Testing sites	Geomin rotated factor loadings			
	1	2	3	4
Abdomen, right upper	0.832*	0.009	0.046	−0.049
Abdomen, right middle	0.827*	0.025	−0.070	0.071
Abdomen, right lower	0.636*	−0.020	−0.036	0.165
Abdomen, midline, pubis	0.737*	0.017	0.163	−0.123
Abdomen, midline, lower 1/3	0.841*	−0.016	0.145	−0.109
Abdomen, midline, umbilicus	0.791*	−0.012	0.002	0.079
Abdomen, midline, upper 1/3	0.724*	−0.010	0.015	0.062
Abdomen, left upper	0.863*	0.010	−0.009	−0.030
Abdomen, left middle	0.804*	−0.009	−0.033	0.107
Abdomen, left lower	0.678*	−0.006	−0.060	0.234
Latissimus dorsi, left	0.714*	0.015	0.109	−0.101
Latissimus dorsi, right	0.599*	0.017	0.157	0.059
Inguinal ligament, right	0.560*	0.043	−0.009	0.157
Inguinal ligament, left	0.546*	−0.009	0.013	0.240
Vulva 12:00	−0.050	0.781*	0.024	−0.015
Vulva 2:00	0.098	0.769*	−0.079	0.031
Vulva 4:00	−0.045	0.748*	0.004	0.058
Vulva 6:00	−0.006	0.703*	0.150	−0.021
Vulva 8:00	−0.003	0.767*	0.172*	−0.084
Vulva 10:00	0.024	0.800*	−0.022	0.101
Puborectalis, right	0.033	0.123*	0.691*	0.048
Iliococcygeus, right	0.001	0.088	0.690*	0.071
Obdurator internus, right	0.022	−0.121*	0.688*	0.241*
Puborectalis, left	0.066	0.055	0.809*	−0.082
Iliococcygeus, left	−0.027	0.058	0.721*	0.067
Obdurator internus, left	−0.028	−0.033	0.659*	0.266*
Uterosacral ligament, right	0.009	−0.037	0.185	0.682*
Uterosacral ligament, left	0.052	0.185*	0.081	0.617*
Adnexal, right	0.073	0.084	0.101	0.635*
Adnexal, left	0.004	0.088	0.054	0.730*

^*ψ*^Factor loadings greater than |0.3| are in bold in the above table.

*Factor loadings are significant at *α* = 0.05.

**Table:** (b)

Factors	1	2	3	4
1	1.000			
2	0.242*	1.000		
3	0.471*	0.525*	1.000	
4	0.507*	0.293*	0.557*	1.000

*Factor correlations are significant at *α* = 0.05.

**Table:** (a)

Classes	Parameters	Log-likelihood	BIC	Entropy	Size of smallest class, *n* (%)
1	28	−6503	13177	N/A	N/A
2	43	−4771	9806	0.952	129 (27.9%)
3	58	−4310	8977	0.908	95 (20.6%)
4	73	−4142	8732	0.906	43 (9.4%)
5	88	−4007	8554	0.924	40 (8.6%)
6	103	−3898	8429	0.931	11 (2.5%)

**Table:** (b)

Classes	Parameters	Log-likelihood	BIC	Entropy	Size of smallest class, *n* (%)
1	12	−6170	12413	N/A	N/A
2	19	−5366	10849	0.989	55 (11.6%)
3	26	−5134	10428	0.984	20 (4.2%)
4	33	−5005	10213	0.980	13 (3.0%)
5	40	−4827	9900	0.949	13 (3.0%)
6	47	−4754	9798	0.933	13 (3.0%)

**Table:** (c)

Classes	Parameters	Log-likelihood	BIC	Entropy	Size of smallest class, *n* (%)
1	12	−7941	15968	N/A	N/A
2	19	−7208	14552	0.984	122 (26.0%)
3	26	−6985	14154	0.962	23 (4.9%)
4	33	−6867	13968	0.959	10 (2.1%)
5	40	−6762	13807	0.933	5 (1.1%)
6	47	−6678	13689	0.930	5 (1.1%)

**Table:** (d)

Classes	Parameters	Log-likelihood	BIC	Entropy	Size of smallest class, *n* (%)
1	8	−4657	9364	N/A	N/A
2	13	−4188	8456	0.902	101 (22.1%)
3	18	−4073	8257	0.899	66 (14.2%)
4	23	−3968	8078	0.851	56 (12.1%)
5	28	−3872	7916	0.872	31 (6.8%)
6	33	−3789	7780	0.846	19 (4.2%)

BIC: Bayesian Information Criterion.

**Table:** (a)

Testing sites	AUC	Optimal threshold	Specificity			Sensitivity		
			Median	95% CI		Median	95% CI	
Left and right upper abdomen	0.966	1	0.8304	0.7887	0.8690	0.9535	0.9147	0.9845
Umbilicus, pubis, Pfannestiel incission, and above the pubis	0.947	3	0.8839	0.8482	0.8839	0.8605	0.7984	0.9147
**Left and right middle abdomen**	**0.974**	**2**	**0.9464**	**0.9196**	**0.9702**	**0.9070**	**0.8527**	**0.9535**
Between the iliac crest and the lower costal margin*	0.921	1	0.8378	0.7988	0.8679	0.9070	0.857	0.9535
Left and right lower abdomen	0.951	1	0.8006	0.7560	0.8423	0.9375	0.8906	0.9766
Left and right inguinal ligament*	0.897	2	0.8269	0.7851	0.8657	0.7969	0.7266	0.8672

*Significantly different from best pair. The best pair or best examination site in each region is marked in bold.

**Table:** (b)

Testing sites	AUC	Optimal threshold	Specificity			Sensitivity		
			Median	95% CI		Median	95% CI	
Vulva 12:00*	0.866	2	0.7636	0.6545	0.8727	0.9330	0.9067	0.9569
Vulva 2:00*	0.844	1	0.7273	0.6000	0.8364	0.9211	0.8947	0.9450
Vulva 4:00*	0.854	1	0.7636	0.6545	0.8727	0.8517	0.8158	0.8852
Vulva 6:00	0.916	1	0.9091	0.8182	0.9818	0.8158	0.7775	0.8517
Vulva 8:00	0.930	3	0.8364	0.7273	0.9273	0.9402	0.9163	0.9617
**Vulva 10:00**	**0.954**	**2**	**0.9455**	**0.8727**	**1.0000**	**0.9187**	**0.8923**	**0.9450**

*Significantly different from best site. The best pair or best examination site in each region is marked in bold.

**Table:** (c)

Testing sites	AUC	Optimal threshold	Specificity			Sensitivity		
			Median	95% CI		Median	95% CI	
Left and right obdurator internus	0.932	10	0.8968	0.8643	0.9292	0.8407	0.7699	0.9027
**Left and right puborectalis **	**0.955**	**6**	**0.8703**	**0.8357**	**0.9049**	**0.9262**	**0.8770**	**0.9672**
Left and right Iliococcygeus	0.948	6	0.8407	0.7994	0.8791	0.9292	0.8761	0.9735

*Significantly different from best pair. The best pair or best examination site in each region is marked in bold.

**Table:** (d)

Testing sites	AUC	Optimal threshold	Specificity			Sensitivity		
			Median	95% CI		Median	95% CI	
**Left and right uterosacral ligament **	**0.991**	**8**	**0.9635**	**0.9438**	**0.9803**	**0.9406**	**0.8911**	**0.9802**
Left and right adnexal*	0.963	9	0.8986	0.8676	0.9296	0.9100	0.8500	0.9600

*Significantly different from best pair. The best pair or best examination site in each region is marked in bold.
